# Prediction of Organic–Inorganic Hybrid Perovskite Band Gap by Multiple Machine Learning Algorithms

**DOI:** 10.3390/molecules29020499

**Published:** 2024-01-19

**Authors:** Shun Feng, Juan Wang

**Affiliations:** 1 Xi’an Key Laboratory of Advanced Photo-Electronics Materials and Energy Conversion Device, School of Electronic Information, Xijing University, Xi’an 710123, China; 2108540002027@stu.xijing.edu.cn; 2 Shaanxi Engineering Research Center of Controllable Neutron Source, School of Electronic Information, Xijing University, Xi’an 710123, China

**Keywords:** organic–inorganic hybrid perovskite, band gap prediction, machine learning model, XGBoost algorithm

## Abstract

As an indicator of the optical characteristics of perovskite materials, the band gap is a crucial parameter that impacts the functionality of a wide range of optoelectronic devices. Obtaining the band gap of a material via a labor-intensive, time-consuming, and inefficient high-throughput calculation based on first principles is possible. However, it does not yield the most accurate results. Machine learning techniques emerge as a viable and effective substitute for conventional approaches in band gap prediction. This paper collected 201 pieces of data through the literature and open-source databases. By separating the features related to bits A, B, and X, a dataset of 1208 pieces of data containing 30 feature descriptors was established. The dataset underwent preprocessing, and the Pearson correlation coefficient method was employed to eliminate non-essential features as a subset of features. The band gap was predicted using the GBR algorithm, the random forest algorithm, the LightGBM algorithm, and the XGBoost algorithm, in that order, to construct a prediction model for organic–inorganic hybrid perovskites. The outcomes demonstrate that the XGBoost algorithm yielded an MAE value of 0.0901, an MSE value of 0.0173, and an R^2^ value of 0.991310. These values suggest that, compared to the other two models, the XGBoost model exhibits the lowest prediction error, suggesting that the input features may better fit the prediction model. Finally, analysis of the XGBoost-based prediction model’s prediction results using the SHAP model interpretation method reveals that the occupancy rate of the A-position ion has the greatest impact on the prediction of the band gap and has an A-negative correlation with the prediction results of the band gap. The findings provide valuable insights into the relationship between the prediction of band gaps and significant characteristics of organic–inorganic hybrid perovskites.

## 1. Introduction

The advancement of materials constitutes a significant catalyst for contemporary scientific development and technological innovation. Rapid advancements in machine learning techniques across various domains have paved the way for the discovery and logical design of materials [1]. Perovskite compounds have gained considerable attention in recent years because of their exceptional properties, establishing them as a vital group of compounds in the field of materials science. Perovskite encompasses a wide range of compounds that possess an identical crystal structure to CaTiO_3_. The general molecular formula for perovskite compounds is ABX_3_, and they derive their name from the Russian geologist Perovski, who initially discovered and defined the perovskite structure [2]. Perovskite materials demonstrate characteristic ferroelectric behavior and have the ability to display a saturated copolarization effect. This phenomenon occurs due to charge-hole separation, resulting in ferroelectricity. The ferroelectric properties of perovskites are primarily determined by the band gap structure [3]. The band gap represents the energy difference between the conduction band and the valence band [4]. Organic–inorganic hybrid perovskite, a derivative of cubic perovskite (ABX_3_), possesses a structure that combines organic and inorganic components. A and B represent two cations, while X represents an anion. Generally, this structure exhibits a cubic or octahedral arrangement [5]. Organic–inorganic hybrid perovskite is a novel type of molecular composite crystal material, formed through the self-assembly of organic and inorganic molecules. The perovskite structure, composed of inorganic metal halides, provides an organized network for carrier transport and enhances the mechanical and thermal stability of the hybrid material. At the same time, the organic components enhance the distinctive optoelectronic functionalities and facilitate the formation of large area films effortlessly [6]. Complex and diverse in their chemical composition and configuration, organic–inorganic perovskite materials exhibit a nonlinear correlation between their structure and properties, rendering conventional theoretical calculations inadequate for their prediction. It has been possible to predict the electronic structure and photoelectrical properties of organic–inorganic perovskite materials using first-principles calculations [7]. However, the calculations are enormous. Not only are machine learning prediction models capable of increasing prediction efficiency, but they can also efficiently extract the implicit structure–performance relationship from massive datasets. Prior to incorporating organic–inorganic perovskite materials into commercial products, it is imperative to identify the critical parameters and optimization strategies pertaining to the fabrication procedure. Machine learning has the capability to analyze substantial volumes of trial data, detect influential factors, and conduct sensitivity analyses. Traditional empirical rules encounter difficulties in elucidating the intricacies of organic–inorganic perovskites. However, machine learning has the capability to autonomously acquire significant rules from extensive datasets, thereby facilitating a more profound comprehension of physical mechanisms. The increasing variety of organic–inorganic perovskite materials necessitates intelligent screening and design; machine learning offers a viable approach to accomplish this. In addition to advancing the study of organic–inorganic perovskite materials from a variety of vantage points, machine learning requires a substantial amount of experimental and computational data to serve as its learning foundation [8,9,10].

In 2018, Lu et al. [11] proposed a fast target-driven method combining machine learning and density functional theory calculations to find stable lead-free hybrid organic–inorganic perovskites. The speed at which this method predicts the band gap value is significantly faster than density functional theory calculations, and the accuracy of its predictions is comparable to that of density functional theory. In 2019, Wu et al. [12] also used the same fast target-driven method to find perovskites suitable for photovoltaic applications from a space two orders of magnitude larger than before (230,808 candidate substances), and they screened 38,230 potentially stable substances based on stability factors and octahedral factors. GBR, SVR, and KRR models were integrated to obtain reasonable prediction results, and 132 nontoxic and stable perovskites were selected as photovoltaic candidates. In 2021, Gao et al. [13] combined machine learning and density functional theory calculations to establish a database of 745 inorganic double perovskites and selected 13 characteristic materials to describe them. The model was trained using the eXtremeGradient Boosting Regression (XGBR) algorithm. Out of a total of 5796 structures, 863 were deemed potentially stable based on the stability factor and octahedral factor criteria. Two nontoxic inorganic double perovskites, namely Na_2_MgMnI_6_ and K_2_NaInI_6_, were screened based on their suitable direct band gaps with the exclusion of conductive precious metals, as predicted by their band gap values. In 2022, Chen et al. [14] combined machine learning, large-scale screening, and density functional theory calculations to screen materials suitable for photovoltaic applications from 77,800 double hybrid organic–inorganic perovskites (DHOIPs). DFT calculations were performed on five models that were developed to evaluate 117 kinds of DHOIPs that contained bromine according to their stability, nontoxicity, charge neutrality, and suitable band gap (1.5–3.0 eV). The average absolute error between the first-principles calculation and the experiment, however, is approximately 0.5 eV; therefore, experimental verification of the screening results is still required.

Unsupervised learning methods offer various applications in perovskite research, providing valuable assistance to researchers in uncovering hidden patterns, reducing dimensionality, extracting features, detecting anomalies, and generating new samples. Techniques such as principal component analysis (PCA) and autoencoders, which are frequently employed in unsupervised learning, are highly efficient in reducing data dimensionality and extracting the most representative features from high-dimensional data [15]. This is especially advantageous for selecting features and visualizing data in perovskite material research, providing essential assistance for studying and applying perovskite materials.

In 2022, Rajan’s team [16] employed unsupervised learning methods to perform toxicological screening of amine compounds in transition metal organic–inorganic doped lithium ion batteries. They collected amine compounds from relevant literature and augmented the compound database by conducting searches based on structural similarity. The amine compounds were categorized into primary groups including aliphatic amines, heterocyclic amines, and anilines, which were then further subdivided into different subcategories. The compounds were encoded using the MHFP6 fingerprint, and the UMAP algorithm was utilized to reduce dimensionality and generate a 2D spatial distribution map called the “amine map.” To obtain data on the biological activity testing of different amine compounds, information from the PubChem database was collected. The toxicity level was quantified using the hit rate. The toxicological data were visualized on the amine maps to observe toxicological relationships among various structural categories and identify less toxic alternative amine compounds. The study presented a compilation of ten amine compounds, both currently available and potential, that are deemed to be safe from a toxicological standpoint and exhibit well-balanced physical characteristics. This compilation can be used as a point of reference when designing synthesis processes. In addition, an artificial intelligence framework was implemented to monitor complex relationships between structure, function, and toxicity, offering database assistance for the investigation of novel amine compounds. Nevertheless, the article suggests specific guidelines for classifying subcategories, and it is yet to be determined if these guidelines can be standardized and quantified to ensure consistent and accurate classification. Additionally, the test results were obtained from the PubChem database, and it is imperative to take into account the potential effects of varying test conditions on the ratio. In summary, this study employed artificial intelligence to methodically assess the toxicological levels of amine compounds in transition metal organic–inorganic lithium ion batteries. The findings provide valuable information for safe synthesis and establishing an amine compound database platform. In 2023, Yuan-Bin She’s group [17] employed transfer learning and data augmentation models to effectively predict the band gaps of metal organic framework materials (MOFs) containing porphyrins. The scarcity of training data specifically tailored for porphyrin-based MOFs (PMOFs) posed a significant challenge in directly applying deep learning techniques. In order to tackle this problem, Yuan-Bin She’s group utilized a graph neural network pretrained on a large dataset to acquire foundational knowledge. Additionally, the volume of PMOF data was expanded through data augmentation techniques such as rotation and mirroring, while other MOF materials were extracted as auxiliary training data. The results showed that accurate band gap predictions were achieved by the graph neural network model, which utilized transfer learning pretraining along with PMOF data augmentation, when comparing the training effects of different strategies. This approach successfully tackled the issue of achieving convergence in deep learning when dealing with limited sample sizes.

In the screening of perovskite materials, machine learning has the capability to rapidly predict band gap values and aid in screening candidates with stability and appropriate band gaps. Due to the limitations of the data and characteristics, additional experimental validation and theoretical analysis are required to confirm the accuracy and viability of these predictions. In this paper, the prediction model of organic–inorganic hybrid perovskite was established by using the Gradient Boosting Regression (GBR) algorithm, the random forest algorithm, the Light Gradient Boosting Machine (LightGBM) algorithm, and the eXtreme Gradient Boosting (XGBoost) algorithm, respectively, and the band gap was predicted. Concurrently, the predicted results were analyzed using the SHAP model interpretation method in order to comprehend the relationship between the predicted band gap and important characteristics of organic–inorganic hybrid perovskites.

## 2. Results and Discussion

### 2.1. Feature Engineering

#### 2.1.1. Feature Processing

Exploratory data analysis is employed to perform data preprocessing on the dataset in this study. MinMaxScaler is utilized to normalize and standardize the newly created dataset [18]. Furthermore, the dataset is mapped to a fixed interval, which eliminates the impact of feature magnitude variation, standardizes all features to the same magnitude, and enables reverse mapping back to the original data. The utilization of MinMaxScaler has the potential to optimize machine learning models:

(1) The minimum value min and maximum value max for each feature are found in the training set.

(2) For each sample, the normalization result of its eigenvalue X is calculated, as shown in Formula (1):

(1) $$X_{normalize}=(x-min)/(max-min)$$

(3) The value of each feature is mapped to the interval [0,1].

The band gap characteristics are visually analyzed by using boxplots and density maps. The median, quartile, minimum, maximum, and outliers of the data, as well as anomalies such as skewness, can be observed.

**Figure 1:**
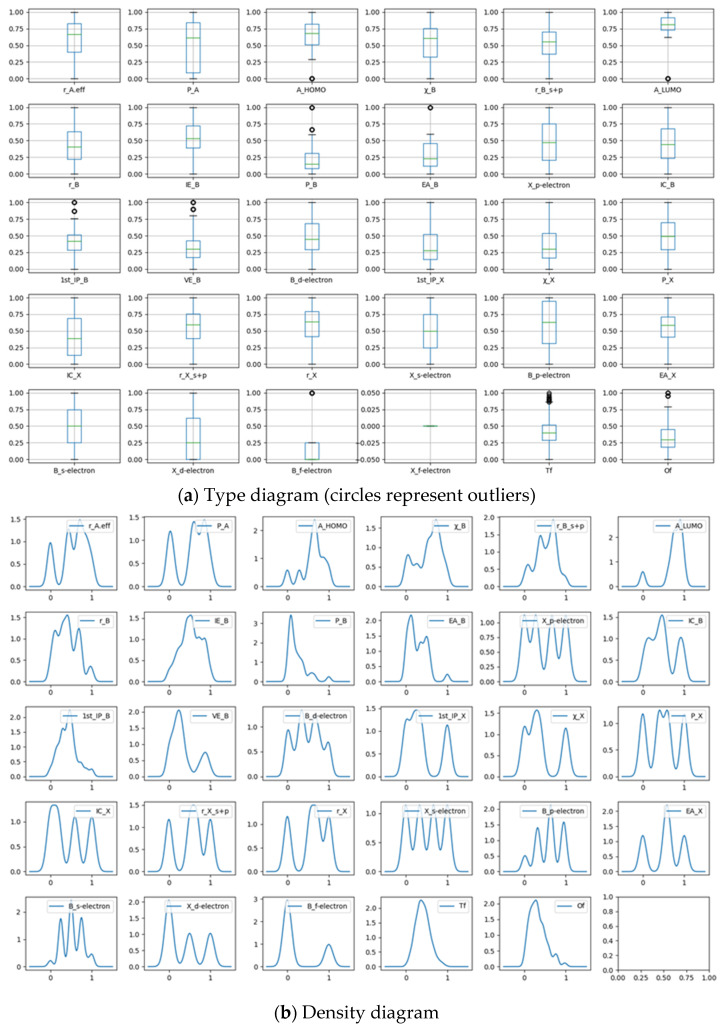
Type diagram (**a**) and density diagram (**b**) of band gap feature box.

This work utilizes a range of visual analysis techniques, such as boxplots and density plots, to gain insights into the data. These techniques provide significant insights into the dataset, such as the median, quartiles, minimum and maximum values, as well as the identification of outliers and skewness. To facilitate comprehensive analysis, the coordinate axes are standardized, and all original features are consolidated into one figure to facilitate comprehensive analysis. In Figure 1a, the horizontal lines above and below the boxplot represent the upper and lower limits of the data, respectively. This plot facilitates the evaluation of the similarity of distributions among various features and the identification of outliers. Outliers are represented by circles that exceed these limits. The X_f-electron eigenvalue serves as an exemplar of an outlier characteristic. Figure 1b displays a density plot of all features, where the X-axis typically represents the value range or feature values and the Y-axis represents the corresponding density. Density plots enable the visualization of the density or frequency of data within specific value ranges. The height of the density value on the Y-axis indicates the density of the data within the corresponding value range on the X-axis. By analyzing Figure 1b, one can gain a more comprehensive comprehension of the distribution of data for each characteristic and effectively assess the similarity of statistical attributes among various features. The density curve depicted in the plot offers valuable insights into the symmetry, peak location, and alterations in the tails of each characteristic, thereby facilitating comprehension of the distribution’s form. Boxplots and density plots are useful visualization tools for data analysis. They facilitate the examination of distributions, detection of anomalies, and comparison of statistical characteristics among various features. Through the application of these visual analysis techniques, researchers can enhance their comprehension of the dataset and extract significant insights from the data.

#### 2.1.2. Feature Correlation Analysis

By choosing the most pertinent subset of features, feature selection is a method for decreased data dimensionality and enhanced model performance. It can facilitate the elimination of superfluous or redundant features, enhance the interpretability and precision of the model, and improve its ability to generalize. The Pearson correlation coefficient is a method to measure the degree of correlation between two features and is usually used to assess the correlation between two features, and its definition is shown in Formula (2):

(2) $$\rho_{X,Y}=\frac{cov(X,Y)=1/(n-1)\sum_{n}^{i=1}(X_{i}-X^{-})(Y_{i}-Y^{-})}{\sigma_{X}\sigma_{Y}}$$

**Figure 2:**
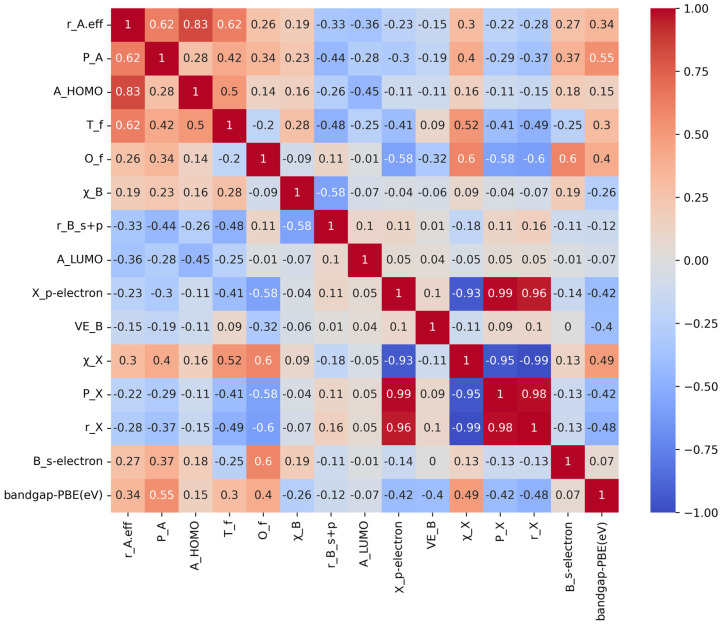
Pearson correlation analysis of band gap features.

Utilizing the Pearson correlation coefficient to assess the correlation between each feature and the target variable, this paper selects features that exhibit a strong correlation with the target variable in order to decrease the number of data dimensions and enhance the performance of the model. In the preliminary screening of band gap data, B_d-electron value, B_p-electron, B_f-electron, IE_B, and 1st_IP_B, these features have no correlation in the range of 0–0.2, so the above features are not considered as features in band gap prediction. Irrelevant features are deleted, and relevant features r_A.eff, P_A, A_HOMO, T_f, O_f, χ_B, r_B_s+p, A_LUMO, X_p-electron, VE_B, χ_X, P_X, r_X, B_s-electron, and bandgap-PBE are retained. A feature Pearson correlation graph is shown in Figure 2. In accordance with the magnitude of the correlation coefficient, the gradient color bar appears to the right. Blue indicates a negative correlation, while red denotes a positive correlation. The paler the color, the weaker the correlation.

**Figure 3:**
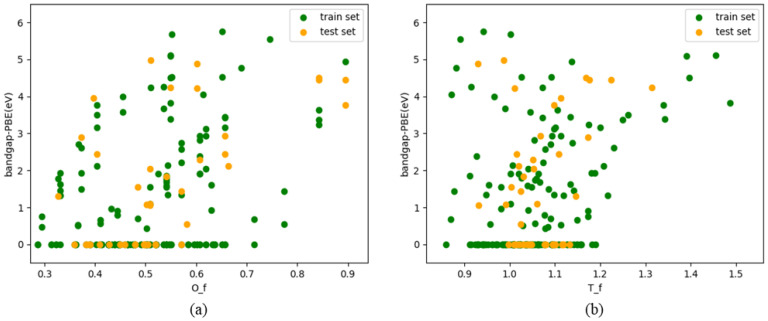
The relationship between the characteristic O_f in (**a**) and the characteristic T_f in (**b**) and the band gap.

As shown in Figure 3, the scatter function was used to illustrate the relationship between the original features and target variables following Pearson correlation analysis. A scatter diagram depicting this relationship was then generated. Regarding the two variables, the diagram illustrates their relationship. Every data point in a scatter diagram corresponds to a sample, with the X- and Y-axis coordinates denoting the respective values of two variables. By observing the distribution and trend of data points in the scatter plot, it is found that the relationship between O_f and T_f and the two variables of band gap has a certain linear relationship.

#### 2.1.3. Feature Screening

**Figure 4:**
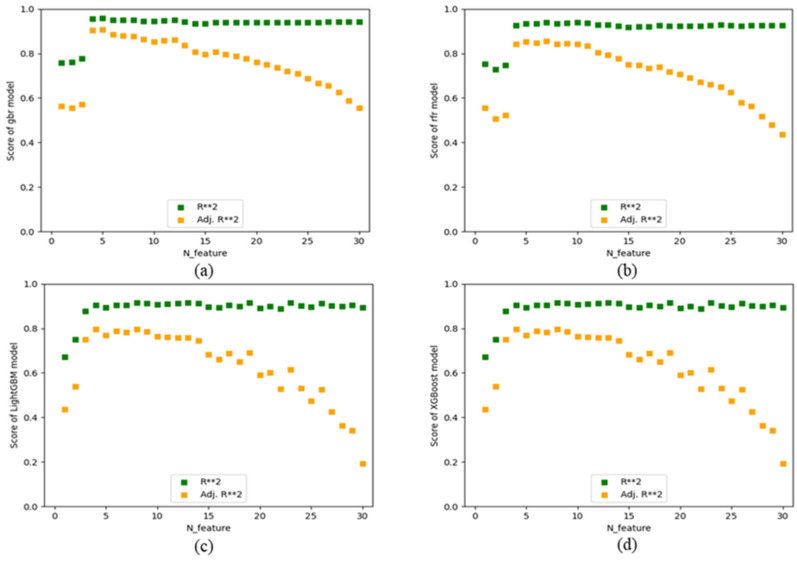
Algorithm feature number score in the bandgap data set (**a**) Feature number score based on GBR (**b**) Feature number score based on RFR algorithm (**c**) Feature number score based on XGBoost (**d**) Feature number score based on LightGBM.

This work thoroughly examines the linear and nonlinear relationships among each feature, based on the original feature distribution. Redundant features with high correlation are eliminated. However, only one of these features is retained; none of the features are purged. In this way, the dimension of the input feature matrix can be reduced, so as to screen out important features in the band gap prediction process of organic–inorganic hybrid perovskite materials. The time it takes to train the dataset is reduced. In this work, four feature selectors are established based on the GBR algorithm, the random forest algorithm, the LightGBM algorithm, and the XGBoost algorithm. The integrated method is used to determine which features are most important for the prediction of target variables, screen the best number of features and feature sets suitable for the model, and screen the worst features each time by different methods. As shown in Figure 4, the scores of the number of features in each algorithm dataset are respectively shown. The reg_score value of GBR model is 0.958048, and the optimal number of features is 5, namely P_A, r_B_s+p, IE_B, X_p-electron, and VE_B. The optimal reg_score value of the random forest regression model is 0.938438, the number of features is 10, and the feature subsets are r_A.eff, P_A, r_B_s+p, IE_B, P_B, X_p-electron, VE_B, χ_X, P_X, and T_f. In the optimal reg_score value of the XGBoost regression model, 0.915030, the number of features is 13 and the feature subsets are r_A.eff, P_A, χ_B, r_B_s+p, IE_B, P_B, X_p-electron, 1st_IP_B, VE_B, IC_X, r_X_s+p, T_f, and O_f. In the best reg_score value of the LightGBM regression model, 0.958756, the number of features is 17. The feature subsets are r_A.eff, P_A, χ_B, r_B_s+p, A_LUMO, IE_B, P_B, EA_B, X_p-electron, 1st_IP_B, VE_B, 1st_IP_X, P_X, IC_X, r_X_s+p, r_X, and X_s-electron.

Complex physical and chemical properties will influence the feature screening stage of organic–inorganic hybrid perovskite band gap prediction, and some physical and chemical properties have intricate interaction relationships with one another. Examination of this relationship may prove inadequate when these features are considered in isolation; therefore, it may be necessary to select corresponding cross-features. Certain physicochemical factors exhibit a nonlinear correlation with the band gap, rendering linear attributes inadequate for their description. In such cases, alternative kernel functions or nonlinear transformations are required. Nonetheless, certain physical and chemical parameters that may influence the band gap may have multimodal distributions; therefore, a mixed model must be considered. In conclusion, the introduction of additional features and noise factors by complex physicochemical properties complicates feature screening. In practice, the aforementioned considerations must be in equilibrium in order to identify the most beneficial attributes for the model.

### 2.2. Model Prediction Results

**Figure 5:**
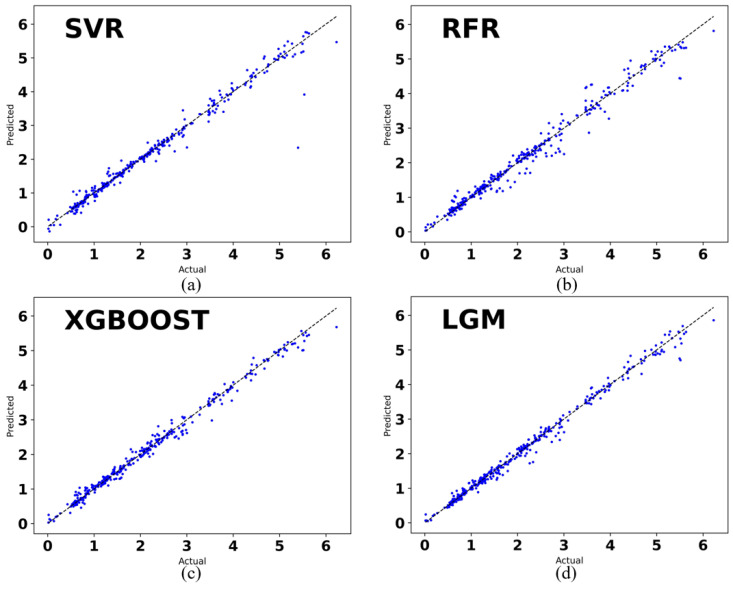
Band gap regression model based on machine learning algorithm. (**a**) Fitting graph based on SVR algorithm. (**b**) Fitting graph based on RFR algorithm. (**c**) Fitting graph based on XGBoost algorithm. (**d**) Fitting graph based on LightGBM algorithm.

Four machine learning algorithms, SVR, RF, XGBoost, and LightGBM, are used to train the training set. In Figure 5, the horizontal coordinate is the true value, which is the organic–inorganic hybrid perovskite band gap calculated by DFT, and the vertical coordinate is the band gap predicted by the machine learning algorithm. A comparison between the predicted and calculated values of the organic–inorganic hybrid perovskite band gap using four machine learning algorithms (SVR, RF, XGBoost, and LightGBM) and DFT yields a linear relationship resembling y = x, as illustrated in the figure. In predicting the band gap value of an organic–inorganic hybrid perovskite, it is possible to conclude that these four algorithms suit the data well. Regression model evaluation performance results are presented in Table 1. The XGBoost algorithm and the LightGBM algorithm have cross-validation R^2^ values of 0.991310 and 0.990867, respectively, suggesting that these two models do not exhibit overfitting or underfitting effects. In contrast, the SVR algorithm has the lowest cross-validation R^2^ of the four models at 0.973959. Certain critical hyperparameters of the SVR algorithm require additional tuning. In comparison to the SVR algorithm and the RFR algorithm, the XGBoost algorithm and LightGBM algorithm produce a prediction model with a significantly higher degree of accuracy.

The XGBoost and LightGBM algorithms are highly potent and extensively employed frameworks for gradient boosting. XGBoost employs regularization strategies such as L1 and L2 regularization to manage the complexity of the model and reduce the likelihood of overfitting. This regularization enhances the model’s ability to effectively generalize to unfamiliar data. Additionally, XGBoost employs an exhaustive sample splitting strategy, considering all possible split points to find the best split, enabling it to capture complex data relationships effectively. Conversely, LightGBM employs a histogram-based algorithm to split samples by discretizing feature values. Although there may be some instances where this approach leads to a loss of information, it typically yields efficient and rapid splitting decisions. XGBoost is specifically designed to efficiently utilize parallel computing, enabling it to effectively leverage multicore processors and distributed computing environments. The ability to parallelize enhances the speed of the model training process, particularly for extensive datasets. Although XGBoost may have certain advantages over LightGBM, the decision between the two models should take into account factors such as the characteristics of the dataset, the complexity of the problem, and the specific needs of the application. In different scenarios, LightGBM may be more suitable, and in some cases, it can outperform the XGBoost algorithm. Thoroughly assessing the attributes of the problem and the dataset is crucial in order to identify the most suitable model for a specific task. Both XGBoost and LightGBM possess formidable capabilities, and the selection between them should be predicated upon a comprehensive comprehension of the particular prerequisites and attributes of the given problem.

Upon analyzing the band gap prediction results of the XGBoost model on the test set, we observed a comparison with the prediction data, which is presented in Table 2. The discrepancy between the predicted and actual values of the majority of machine learning algorithms for organic–inorganic hybrid perovskite can be constrained to a range of 0.000758–0.076244. The range of accurate values is determined using DFT. The organic–inorganic hybrid perovskite compound composed of CH_3_CH_2_NH_3_ at the A position, Sn at the B position, and I at the X position has a large error. Feature screening shows that the octahedral factor and the tolerance factor have the least impact on band gap predictions. However, the presence of organic molecules may mask the effects of octahedral factors and tolerance factors on the crystal structure.

### 2.3. SHAP Interpretation of Bandgap Models Performance Evaluation of Regression Models

The SHapley Additive exPlanations (SHAP) method explains the model’s prediction results by analyzing the contribution of each feature [19]. In addition to explaining the local model, this method can also provide an explanation of the global model and the extent to which each feature contributes to it. It makes up for the shortcomings of the above two methods. SHAP values utilize the concept of Shapley values from game theory to assign feature importance and explain the model’s predictions for each sample. SHAP values provide a way to analyze the contribution of each feature in predicting the target variable (such as the band gap in the example). By examining SHAP values, you can identify key features that have the most significant impact on model predictions. This understanding helps to explain how models leverage different features to make predictions. Additionally, SHAP values can help to verify or discover the impact of features related to domain knowledge. SHAP values provide a valuable tool for feature importance analysis and model prediction interpretation, allowing researchers to gain insight into the impact of features on target variables and potentially validate or discover domain knowledge.

The SHAP method can effectively elucidate tree regression prediction results, which were used to consider the interaction between features and make up for the lack of explanation of the model prediction results. The SHAP method can be employed to aid the tree model in handling missing values, thereby enhancing the accuracy and resilience of model interpretation. By quantifying the global and local impacts of each feature and offering precise numerical justifications, it aids in comprehending the predictive capability of the model and the significance of the features. In this work, the SHAP model interpretation method can be utilized to identify the features that exert the most significant influence on the band gap prediction task. The determination of whether the characteristic has a positive or negative impact on the band gap can be made by examining the magnitude and polarity of the SHAP value. A higher positive SHAP value signifies a greater contribution of the feature to the increase in the band gap, whereas a higher negative SHAP value signifies a greater contribution of the feature to the reduction of the band gap. By doing so, we can discern the characteristics that are crucial to the band gap. This explanation facilitates our comprehension of how the model generates predictions by analyzing input features and enables us to identify and comprehend the crucial features that impact the band gap.

**Figure 6:**
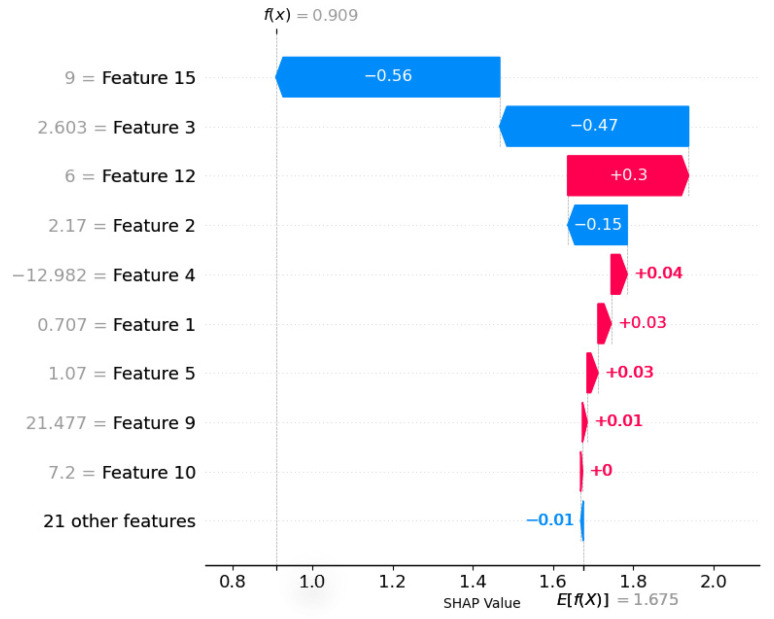
Correlation between band gap prediction and input features. (Red represents positive and Blue represents negative).

The prediction results of the feature dataset and model are shown in Figure 6. In the feature screening stage, the input feature subsets to establish the prediction model for the best XGBoost algorithm are r_A.eff, P_A, χ_B, r_B_s+p, IE_B, P_B, X_p-electron, 1st_IP_B, VE_B, IC_X, r_X_s+p, T_f, and O_f. Despite the fact that A is regarded as an organic cation, atomic parameters continue to govern the inorganic portion of organic–inorganic hybrid perovskites. However, the organic molecular and atomic characteristics are seldom encountered due to the complexity of the organic cation at the A site. The SHAP method decomposes the contribution of each feature into the sum of the contribution of different feature subsets, which are defined by the concept of Shapley values. It is presented with a bar plot, where the contribution of each feature is represented by a bar and arranged in order of the contribution from largest to smallest. Figure 6 presents a ranking of the ten most significant feature SHAP values, arranged in descending order of feature importance. The value of the feature is inversely proportional to the positivity of the prediction result, with red denoting positive influence. A higher value of this feature signifies negative effects; that is, the prediction results are more skewed toward the negative class. In the model’s prediction results, the horizontal coordinate signifies the SHAP value, which denotes the proportion of each feature’s contribution. A negative value signifies a negative contribution of the feature to the forecast result, whereas a positive value signifies a positive contribution of the feature to the prediction result. The ordinate signifies the feature name associated with the SHAP value on the abscissa, which is the name of each individual feature. As illustrated in Figure 6, the most critical characteristic for the band gap prediction model is the highest molecular orbital, and its value exhibits a negative correlation with the model. Similarly, χ_B demonstrates a positive correlation with the band gap prediction model. The figure struggles to depict the correlation between other features and the band gap prediction due to the diminishing significance of the Shapley feature. This demonstrates that the characteristics with the highest SHAP value importance are more influential in predicting the band gap, thereby improving the model’s interpretability.

## 3. Data and Methods

### 3.1. Data Sources

Experimental literature and open-source material gene databases provided the information for the organic–inorganic hybrid perovskites utilized in this study. Lawrence Berkeley National Laboratory and Carnegie Mellon University have collaborated to establish the Materials Project, an open access materials database. Its principal objective is to expedite the process of material discovery and facilitate the development of novel materials [20]. The Materials Project database collects and calculates the structure and properties of more than 15,000 kinds of materials based on the data obtained from first-principles calculation. All the data are available for unrestricted distribution and use, providing a platform for team collaboration. New material calculations and database expansion are ongoing on the part of the project team. Recognized and utilized by the materials science community, the Materials Project offers platforms and data resources that are extremely beneficial to materials science research. It facilitates advancements in the materials field and reduces the time between experimental discovery and commercial application. By performing the following operations on the initial dataset: data cleansing, removing rows containing duplicate values and absent values for preliminary screening purposes, and obtaining a total of 201 datasets from the initial dataset. Each element in the new dataset containing every possible combination of A, B, and X is violently combined to produce 1409 data points. There is hash function-based deletion of extant combinations and row deletion of organic–inorganic hybrid perovskite compounds. The output is 1208 data points comprising solely unidentified combination compounds with their corresponding attributes.

### 3.2. Machine Learning Regression Model

#### 3.2.1. Gradient Lift Regression (GBR)

The Gradient Boosting Regression algorithm (GBR), a supervised learning algorithm, is widely used in machine learning, especially in regression problems [21]. The GBR improves the model by iteratively learning multiple base learners. The basic idea of the GBR is that the new base learner fits the residual of the previous base learner. The algorithm flow is as follows:

Let the training dataset be $\left\{\left(x1,\;\;y1\right),\;\;\left(x2,\;\;y2\right),\;\;\cdots ,(xn,\;\;yn)\right\}$, where xi∈Rm, $y_{i}\in R$, and m is the characteristic number.

(1) For initialization, Formula (3) is as follows:

(3) $$f_{0}\left(x\right)=argmin_{\gamma }\sum_{i=1}^{n}L\left(y_{i},\;\gamma \right)$$

(2) For each iterative step t = 1, 2, ⋯, T, the negative gradient is calculated, as shown in Formula (4). The residual is fitted into a regression tree, the leaf node region $R_{jt}$ of the t th tree is obtained, [j=1, 2,  ⋯], and the output value of each leaf node is calculated, as shown in the formula. The model is then updated, as shown in Formulas (4)–(6), to return the final model: $f_{T}(x)$.

(4) $$r_{it}=-[\frac{\partial L(yi,\;\;f(xi))}{\partial f(xi)}{]}_{f(x)=ft-1(x)}$$

(5) $$\gamma_{jt}=argmin_{\gamma }\sum_{x_{i}\in R_{jt}}L\left(y_{i},\;f_{t-1}\left(x_{i}\right)+\gamma \right)$$

(6) $$f_{t}(x)=f_{t-1}(x)+\sum_{j=1}^{J}\gamma_{jt}I(x\in R_{jt})$$

where L(y,  f(x)) is the loss function and I(x∈Rj) is the indicator function. In the GBR algorithm, the square error loss function is usually used.

#### 3.2.2. Random Forest (RF)

Random forest is an algorithm composed of decision trees proposed by BREIMAN [22] in 2001. RF, an ensemble learning bagging method, executes machine learning tasks through the construction of combinations of numerous decision trees, which are typically applied for classification and regression. An advantage of ensemble learning is that it offers superior prediction performance compared to a singular estimator, in addition to enhancing the universality and robustness of the former. An additional characteristic of ensemble learning is its straightforward parallelizability. Bagging is a method of general integrated learning. Designated for decision trees, random forests are a specialized form of bagging. Random forest enhances the bagging effect by decreasing the correlation of the base learner through the introduction of randomness at each node.

The construction process of random forest is mainly divided into the following two parts.

The first part involves constructing a decision tree. Numerous decision trees were generated by employing bootstrap sampling and selecting features at random. By reducing the likelihood of overfitting, this approach enhances the ability to generalize.

The second part is the integration of multiple decision trees. By voting or averaging the prediction results of each decision tree, higher precision and more stable final prediction can be obtained, which realizes the ensemble learning effect of random forest.

#### 3.2.3. Support Vector Regression (SVR)

Support vector machine (SVM) is a supervised learning algorithm [23], which belongs to a type of sparse learning and only relies on support vectors to make predictions. The operation of support vector machines consists of locating an optimal hyperplane within a given dataset that partitions each point into distinct classes of data. By maximizing the distance between the data concentration point and the hyperplane, one can determine the optimal hyperplane. One can assign the greatest distance from the hyperplane to the point that is closest to it (the support vector). One can consider these support vectors to be entities that characterize the hyperplane. Adjusting the position of the hyperplane and mapping the data to a high-dimensional feature space accomplish this. SVC is support vector classification, while SVR is support vector regression. These are two distinct models of machine learning. Classification problems are the primary focus of SVC, which excels at both linear and nonlinear classification in high-dimensional spaces. To predict a continuous value, SVR is primarily used in regression problems; Formula (7) is as follows:

(7) $$y(x)=w^{T}\Phi (x)+b$$

where w is the weight vector, b is the bias, and the hyperplane is uniquely determined by w and b.

The hyperplane and classification decision function are separated as outputs, and the penalty parameter C>0 is selected. At 0, one can construct and solve the convex quadratic programming problem. C>0 is called the penalty parameter, and the greater the value of C, the greater the penalty for classification. Formula (8) is as follows:

(8) $$\min_{\alpha }\frac{1}{2}\sum_{i=1}^{N}\sum_{j=1}^{N}\alpha_{i}\alpha_{j}y_{i}y_{j}(xi\cdot xj)-\sum_{i=1}^{N}\alpha_{i}$$

At the same time that $\sum_{i=1}^{N}\;\alpha_{i}y_{i}=0$, $0\leq \alpha_{i}\leq C,i=1,2,\ldots ,N$, the optimal solution $\alpha^{*}={\left(\alpha_{1}^{*},\alpha_{2}^{*},\ldots ,\alpha_{N}^{*}\right)}^{T}$.

Finally, the classification decision function Formula (9) is obtained, which is as follows:

(9) $$f(x)=sign(w*\cdot x+b^{*})$$

One of the components of $\alpha^{*}$, $\alpha_{j}^{*}$, satisfies the condition $0<\alpha_{j}^{*}<C$; $b^{*}=y_{j}-\sum_{i=1}^{N}\;\alpha_{i}^{*}y_{i}K(x_{i},\;\;x_{j})$.

#### 3.2.4. Extreme Gradient Lift Algorithm (XGBoost)

The XGBoost [24] algorithm is an algorithm implementation of boosting architecture, which also conforms to the model function, and the output of the model can be expressed as the sum of the output of K weak learners. The fundamental concept is to continuously generate new trees, with each tree acquiring knowledge by comparing its output to the desired value, thereby mitigating the model’s bias. Formula (10) displays the outcome of the ultimate model result. In other words, the model’s predictions for a sample are equal to the sum of the tree results.

(10) $${\hat{y}}_{i}^{\left(t\right)}=\sum_{k=1}^{i}f_{k}(x_{i})={\hat{y}}_{i}^{\left(t-1\right)}+f_{t}(x_{i})$$

where ${\hat{y}}_{i}^{\left(t\right)}$ is the prediction result of sample i after the t iteration, ${\hat{y}}_{i}^{\left(t-1\right)}$ is the prediction result of the former t−1 tree, and $f_{t}(x_{i})$ is the model of the t tree.

The loss function can be expressed by the predicted value ${\hat{y}}_{i}$ and the true value $y_{i}$, and the formula is as follows (11):

(11) $$L=\sum_{i=1}^{n}l(y_{i},{\hat{y}}_{i})$$

where n is the number of samples.

The accuracy of the model’s predictions is assessed through its variance and deviation, with the loss function serving as a representation of the model’s deviation. Routing regular terms into the objective function is imperative in order to avert overfitting when the variance is minimal. As a result, the objective function is composed of the model’s loss function and the regular term that restricts the model’s complexity. The definition of the objective function is as follows (12):

(12) $$Obj=\sum_{i=1}^{n}l(y_{i},{\hat{y}}_{i})+\sum_{i=1}^{t}\Omega (f_{i})$$

Among these, $\sum_{i=1}^{n}\;l(y_{i},{\hat{y}}_{i})$ is the sum of the complexity of all *t* trees and is added to the objective function as a regularization term to prevent the model from overfitting.

Since XGBoost is an algorithm in the boosting family, it follows forward stepwise addition. Taking the model at step *t* as an example, the model predicts the *i*th sample $x_{i}$ with the following Formula (13):

(13) $${\hat{y}}_{i}^{\left(t\right)}={\hat{y}}_{i}^{\left(t-1\right)}+f_{t}(x_{i})$$

where ${\hat{y}}_{i}^{\left(t-1\right)}$ is the predicted value given by the model at step t−1 and is a known constant, and $f_{t}(x_{i})$ is the predicted value of the new model to be added this time. In this case, the objective function can be written as follows in Formula (14):

(14) $$Obj^{\left(t\right)}=\sum_{i=1}^{n}l(y_{i},{\hat{y}}_{i}^{\left(t\right)})+\sum_{i=1}^{t}\Omega (f_{i})$$

The term of regularization is divided. Given the knowledge of the prior tree’s structure, the sum of its complexity can be mathematically represented by the following constant Formula (15):

(15) $$\sum_{i=1}^{t}\Omega (f_{i})=\Omega (f_{t})+constant$$

#### 3.2.5. Lightweight Gradient Lifting Algorithm

On the premise of a decision tree, the Microsoft team devised a lightweight gradient lifting algorithm. This algorithm processes a large number of data instances and features, respectively, using gradient-based unilateral sampling and exclusive feature aggregation, two novel technologies [25]. LightGBM is a machine learning algorithm based on the Gradient Boosting Decision Tree (GBDT). Compared with the traditional GBDT, LightGBM uses some new techniques, such as the histogram-based decision tree algorithm and mutually exclusive feature bundling, to improve the training efficiency and accuracy of the model. In addition to its efficacy in managing extensive datasets, LightGBM has demonstrated commendable performance in numerous machine learning competitions. LightGBM has numerous benefits, including enhanced precision, accelerated training speed, the capacity to process massive amounts of data, and GPU learning support.

Assume that the dataset is $H=\{(x_{i},y_{i}){\}}_{i=1}^{n}$, and use the LightGBM algorithm to find the approximate value $\hat{f}(x)$ of a function f(x). The loss function L(y,f(x)) can be minimized through the function. To judge the quality of the model fitting data, observe the size of the loss function, whose optimization function can be expressed as follows in Formula (16):

(16) $$\begin{array}{c} \hat{f}=\arg\underset{f}{min}\;E_{y,X}L(y,f(x)) \end{array}$$

Meanwhile, the LightGBM model integrates k regression trees to fit the final model, which can be expressed as:

(17) $$f_{k}(x)=\sum_{i=1}^{k}\;f_{t}(H)$$

Indeed, the majority of machine learning tools lack the capability to directly process category features. Instead, they require the implementation of one-shot coding to transform category features into multidimensional 0-1 features, a process that significantly wastes time and space. Nevertheless, it is common knowledge that one-shot coding is not advisable for decision trees, particularly when the category features contain a substantial number of categories. This is due to the subsequent issues that may arise: Asymmetry in sample segmentation will ensue, thereby impeding the decision tree learning process. Beyond the utilization of histogram algorithms and mutually exclusive feature bunching, LightGBM further enhances the performance of the model by employing techniques like Gradient-based One-Side Sampling (GOSS) and Exclusive Feature Bundling (EFB). These methods contribute to improved training efficiency and accuracy. By decreasing the quantity of training data without sacrificing model accuracy, GOSS can speed up training. By preserving the accuracy of the model while decreasing the complexity of the decision tree, EFB can enhance the generalizability of the model. For a wide range of classification and regression issues, LightGBM is an exceptionally potent and effective machine learning algorithm. It has significantly enhanced the performance and computational efficacy of classification tree models, providing machine learning engineers and data scientists with a valuable instrument.

### 3.3. Model Evaluation Index

The performance of regression models can be evaluated using many different metrics [26]. The most common of these are mean squared error, root mean squared error, mean absolute error, and R^2^ (R-squared). MSE measures the sum of squares of the difference between the predicted value and the true value, and RMSE is the square root of MSE. MSE and RMSE usually measure model performance in the same way. RMSE has the advantage that it is proportional to the size of the regression target and is easier to understand. MAE measures the average of the sum of the difference between the predicted value and the true value. The difference between the predicted value and the true value can be determined using both mean squared error and mean absolute error as indicators. The mean absolute error is more sensitive to small values of forecast error, whereas the mean squared error is more sensitive to large values of forecast error. R^2^, representing the degree of linear correlation between the regression value and the true value on a scale from 0 to 1, with higher values indicating that the model is better at explaining changes in the data, is another frequently employed metric that measures how well the model explains changes in the data. In addition to these indicators, there are other indicators that can be used to evaluate the performance of regression models, such as root mean squared error (RMSE), mean absolute percentage error (MAPE), etc. Their Formulas (18)–(20) are as follows:

(18) $$MSE=\frac{1}{m}\sum_{i=1}^{m}(f_{i}-y_{i}{)}^{2}$$

(19) $$RMSE=\sqrt{\frac{1}{m}\sum_{i=1}^{m}\;(f_{i}-y_{i}{)}^{2}}$$

(20) $$R^{2}=1-\frac{\sum_{i=0}^{n-1}\;(y_{i}-f_{i}{)}^{2}}{\sum_{i=0}^{n-1}\;(y_{i}-{\bar{y}}_{i}{)}^{2}}$$

where m is the number of samples; $f_{i}$ is the true value; and $y_{i}$ is the predicted value.

## 4. Conclusions

The objective of this paper is to predict the band gap values of organic–inorganic hybrid perovskites using a machine learning model. Some 201 data points were gathered via literature reviews and open-source databases. Three distinct files were created to store the violent combination by separating the characteristics of bits A, B, and X. Each element was then combined to produce 1409 data points; ultimately, a dataset comprising 1208 data points was generated, which included 30 feature descriptors of unknown combination compounds and all features. Following the preprocessing of the dataset, a Pearson correlation coefficient analysis comparison is performed on the original features, and the features that yield the highest effectiveness are chosen as the feature subset. The GBR algorithm optimizes the partitioning of the acquired dataset into a training set of 9:1. In order to forecast the critical performance of the organic–inorganic hybrid perovskite band gap, various machine learning prediction models were developed. In this step, the optimal hyperparameters for the prediction model are identified via grid search in order to enhance the model’s performance when comparing the results of band gap predictions. Upon conducting a comparative analysis of the evaluation indicators, it is evident that the XGBoost algorithm’s prediction model exhibits the most favorable prediction effect. The MAE value is 0.0901, the MSE value is 0.0173, the R^2^ value is 0.991310, and the model’s prediction error is minimal. These results further suggest that the input features effectively fit the prediction model in comparison to the initial input features and default parameters. When it comes to model selection, model complexity and efficacy are the determining factors. The prediction results of the XGBoost algorithm’s prediction model were analyzed using the SHAP model interpretation method. It was determined that the occupancy rate of ions in the A-position had the greatest impact on the band gap prediction results and was negatively correlated with those results. The SHAP method helps us better understand the prediction process and the importance of characteristics of the model, and to find the key characteristics that affect the band gap value of organic–inorganic hybrid perovskite, so as to improve the interpretability and application value of the model.

Undoubtedly, the limited availability of training data in the field of chemical materials, such as perovskites, can pose difficulties when employing machine learning. To address these limitations, further research should prioritize the following areas. (1) For small sample data enhancement, implement data enhancement techniques like rotation, mirroring, or other transformations to expand the training dataset. By generating additional samples from limited data, this approach helps to increase the size of the training set and improves the model’s generalization ability. (2) For auxiliary training data from similar materials, calculate the properties of other titanate materials that exhibit similarities with perovskites. Utilize material representation learning techniques to extract feature similarities between perovskites and other titanate materials. Integrating more supplementary data during the training process can offer extra information to improve the performance of the model. (3) For multimodal data fusion, if possible, investigate approaches that integrate experimental data, including spectral and electrical characteristics of perovskites, with calculated values and other data sources. By fusing multiple data modalities, the model can benefit from both experimental and computational information, potentially improving the accuracy and robustness of predictions. These research directions aim to overcome the challenges posed by limited training data and enhance the performance of machine learning models for perovskite materials. Researchers can enhance the effectiveness and applicability of machine learning methods in this field by utilizing techniques such as data augmentation, auxiliary data, and multimodal fusion.

## Figures and Tables

**Table 1:** Comparison of performance results of band gap evaluation regression models.

Algorithm	Evaluation Index		
	MAE	MSE	R^2^
SVR	0.1001	0.0518	0.973959
RFR	0.1207	0.0407	0.979509
XGBoost	0.0901	0.0173	0.991310
LightGBM	0.0850	0.0182	0.990867

**Table 2:** Prediction results of XGBoost model on band gap value on test set.

HOIP Serial Number	pbe_bandgap/eV	ml_bandgap/eV	Prediction Error/eV
1	1.544658	0.940840	0.603818
2	0.000000	0.017771	−0.017771
3	0.000000	−0.000758	0.000758
4	3.764700	3.230945	0.533755
5	0.000000	0.030329	−0.030329
6	0.000000	−0.031874	0.031874
7	2.049300	1.686907	0.362393
8	0.000000	−0.008076	0.008076
9	4.238700	4.576898	−0.338198
10	4.499900	4.547920	−0.04802
11	0.000000	−0.028204	0.028204
12	4.445400	3.654031	0.791369
13	4.216821	4.292674	−0.075853
14	2.895200	2.293573	0.601627
15	0.000000	−0.008764	0.008764
16	2.443239	1.891167	0.552072
17	1.085600	1.236205	−0.150605
18	2.113336	1.794438	0.318898
19	0.000000	0.003571	−0.003571
20	0.000000	0.076244	−0.076244

## Data Availability

Data are contained within the article.
